# Involvement of C-peptide in the progression of type 2 diabetes mellitus through triglyceride-centered lipid metabolism

**DOI:** 10.1007/s42000-025-00737-0

**Published:** 2025-11-25

**Authors:** HuiFang Li, ZhaoMing Zhu

**Affiliations:** Department of Nephrology, Hebei Yiling Hospital, Shijiazhuang, Hebei 050091 China

**Keywords:** Fasting C-peptide, Type 2 diabetes, Triglycerides, Disease progression

## Abstract

**Purpose:**

Fasting C-peptide (FCP), which is co-secreted with insulin, plays a critical role in the pathogenesis of diabetic complications as both deficient and excessive secretion are associated with adverse outcomes. This study aimed to determine the relationship between FCP levels and lipid metabolism disorders—particularly those reflected by triglyceride (TG) levels—and related clinical indicators.

**Method:**

A total of 607 patients (median age 63 years) were included and categorized into four groups according to their FCP levels. Pearson’s correlation analysis was performed to evaluate the relationships between FCP and other clinical variables. The area under the receiver operating characteristic curve was used to evaluate the predictive performance of TG, uric acid (UA), and fasting blood glucose levels for elevated FCP. Univariate and multivariate logistic regression analyses were used to identify independent risk factors influencing FCP levels.

**Results:**

The prevalence of diabetic kidney disease was 42.36%. TG levels, TG-glucose index (TyG), and atherogenic index of plasma (AIP) varied significantly among the four groups (*P* < 0.01). FCP showed significant correlations with TG, TyG, AIP, UA, and creatinine (all *P* < 0.001). Stratified analysis indicated that each unit increase in TG or UA was associated with a significant rise in FCP (both *P* < 0.001). Moreover, TG level was identified as an independent risk factor for elevated FCP (*P* = 0.013).

**Conclusion:**

In patients with type 2 diabetes mellitus (T2DM), FCP levels are closely associated with dysregulation of lipid metabolism, characterized by elevated TG levels that may promote target organ damage. These findings suggest that C-peptide supplementation may not be beneficial for delaying the progression of T2DM or its complications.

**Supplementary Information:**

The online version contains supplementary material available at 10.1007/s42000-025-00737-0.

## Introduction

C-peptide, which is co-secreted in equimolar amounts with insulin, is a widely-used biomarker of pancreatic beta (β)-cell function [1]. Studies have shown that C-peptide replacement therapy reduced the severity of various complications associated with diabetes in animal models and patients with type 1 diabetes, including neuropathy, nephropathy, vascular dysfunction and damage, retinopathy, and impaired wound healing [2–4]. However, the role of C-peptide in type 2 diabetes mellitus (T2DM) has yet to be elucidated. Over the past 20 years, research has suggested that C-peptide is a bioactive peptide that influences complications associated with diabetes [5]. In the early stages of T2DM, C-peptide secretion is higher than physiological levels and closely related to insulin resistance (IR). Marx et al. determined through immunohistochemical staining that patients with diabetes exhibited a higher deposition of C-peptide in the intima of the thoracic aorta, which is co-localized with monocytes/macrophages in the arterial intima, than those without diabetes [6]. Animal models have shown that C-peptide binds to signal transduction molecules on the cell membrane surface, subsequently activating downstream signaling pathways that exert antioxidant and anti-apoptotic effects, regulate inflammatory responses, and modulate cell transcription through internalization [7]. Fasting C-peptide (FCP) is a static response of β-cells to plasma glucose concentration. As C-peptide is not affected by hepatic clearance rates, its uptake in the liver is considered negligible. C-peptide levels obtained while a patient is fasting are less affected by confounding factors and are, therefore, easier to standardize. C-peptide has a consistent renal clearance rate, close to the glomerular filtration rate (GFR) and most C-peptide is metabolized by the renal tissue; therefore, its urinary excretion is relatively low [8–10]. At present, most of the potential clinical applications of C-peptide in T2DM have not been supported by conclusive evidence.

Dyslipidemia in patients with T2DM is primarily characterized by disorders related to an elevated triglyceride (TG) level [11]. Hypertriglyceridemia increases the accumulation of free fatty acids in the skeletal muscles, resulting in IR [12]. Additionally, at higher TG levels the breakdown of fat is reduced, leading to a significant increase in the accumulation of circulating chylomicron and very-low-density lipoprotein (LDL) remnants. This increase promotes endothelial dysfunction and inflammation, increased β-cell dysfunction, and cell apoptosis. For decades, increased levels of TG and TG-rich lipoproteins have been widely recognized as risk factors for atherosclerotic cardiovascular disease [13, 14]. The triglyceride-glucose (TyG) index, a combination of TG and fasting blood glucose (FBG), has been identified as a more reproducible biomarker of IR than the homeostatic model assessment for IR (HOMA-IR) [15, 16]. The atherogenic index of plasma (AIP), which is the ratio of TGs to high-density lipoprotein (HDL), is a biomarker for plasma atherosclerosis used to predict the development of cardiovascular events and their associated mortality rates [17]. Elevated AIP is significantly associated with an increased prevalence of both prediabetes and diabetes [18]. This study investigated the relationship between the FCP level and disorders of lipid metabolism—based on TG levels—in patients with T2DM.

## Methods

### Participants

This retrospective study included consecutive patients with T2DM admitted to the Hebei Yiling Hospital Affiliated to Hebei Medical University (Shijiazhuang, Hebei, China) between January 1, and November 30, 2023. The inclusion criteria were as follows: (1) patients diagnosed with T2DM as defined by the World Health Organization or the Chinese Diabetes Society [19] who have undergone treatment for hyperglycemia for >6 months, and (2) age ≥ 40 years [20]. The exclusion criteria were as follows: (1) patients without T2DM, (2) patients with T2DM concomitant with lactic acid poisoning, ketoacidosis, severe infection, or other critical illness (e.g., organ failure, acute myocardial infarction, and cerebral infarction.), and (3) patients for whom laboratory testing data was incomplete. This study was approved by the ethics committee of Hebei Yiling Hospital Affiliated to Hebei Medical University (approval no.: 2024LCKY-014-01) and carried out in accordance with the Declaration of Helsinki. All patients provided written informed consent prior to their participation.

### Clinical and biochemical data collection

Patient clinical information, baseline demographic data, and laboratory data were extracted from the hospital’s electronic medical recording system by trained physicians. Hematological parameters were determined based on patients’ laboratory test results obtained during their first hospitalization, and blood samples were collected in the morning, after each patient had fasted for ≥ 8 h. All blood and urine samples were tested immediately following their collection. Biochemical markers including FBG, TG, LDL-C, UA, CR, and β2-MG were quantified using a fully automated biochemical analyzer (AU5800, Beckman Coulter); FCP and oral glucose tolerance testing were performed using electrochemiluminescence (Roche E601); and coagulation markers including Fib, D-D, and FDP were quantified using an automatic coagulation analyzer (CS-5100; Sysmex Europe GmbH).

### Definitions

Estimated GFR (eGFR) was calculated using the Chronic Kidney Disease Epidemiological Collaborative Formula (CKD-EPI) [21, 22].

$$\mathrm{AIP}=\log10\left(\mathrm{TG}/\mathrm{HDL}-\mathrm C\right)$$

$$\begin{array}{lc}\mathrm{Systematicimmune}-\mathrm{inflamationindex}\left(\mathrm{SII}\right)=\\\mathrm{platelet}\;\mathrm{count}\times\mathrm{neutrophil}\;\mathrm{count}/\mathrm{lymphocyte}\;\mathrm{count}\end{array}$$

$$Triglyceride glucose (TyG) = In(fastingTG[mg/dL]) x FBG [mg/dL]/2)$$

### Statistical analysis

All data were analyzed using SPSS 23.0 software (SPSS Inc., Chicago, IL, USA). The data are expressed as mean ± standard deviation or median with interquartile range for continuous variables and as percentage for categorical variables. One-way analysis of variance was used for intergroup comparisons. Pearson’s correlation analysis was used to evaluate the relationship between C-peptide and other clinical variables. The constructed receiver operating curve (ROC) was used to evaluate the identification performance of higher FCP levels, according to the area under curve (AUC). Univariate and multivariate regression analyses were performed to evaluate risk factors for high FCP levels. Statistical significance was defined as a *P*-value < 0.05.

## Results

### Clinical data

A total of 607 patients (median age, 63 years) were included in this study. The prevalence of DKD was 47.78% (0.6% and 41.76% of patients had an eGFR < 60 mL/min/1.73 m^2^ and UACR > 30 mg/g, respectively). No statistically significant differences in FCP levels were observed between the DKD and non-DKD groups (see Supplementary Material Table 1). Patients were assigned to four groups (FCP 0, 1, 2, and 3) based on FCP level quartiles, from lowest to highest. The differences in TG levels, TyG indices, and AIPs among the four groups were statistically significant (all *P* < 0.01). ALT, UA, LDL-C, Fins, blood glucose, eGFR, and HOMA-IR were significantly higher in the FCP3 group (FCP > 2.5651) than in the FCP0 group (FCP < 1.653). However, β2-MG, GLB, Fib, ACR, ALB, Hcy, GCT, CR, Cysc, and HDL were only significantly different for FCP > 3.591 (FCP3) compared with FCP < 1.653 (FCP0). PT, TT, PLT, and SII did not differ significantly among all FCP groups (Table 1).

**Table 1:** Clinical characteristics according to plasma fasting C-peptide level quartiles

Various	0 group (FCP<1.653ng/ml) *N* = 103	1 group(1.6531–2.6531.565ng/ml) *N* = 112	2 group (2.5651–3.5651.59ng/ml) *N* = 104	3 group >3.591ng/ml *N* = 97
β2-MG(mg/L)	3.789 ± 20.115	2.0358 ± 0.863	2.137 ± 0.903	2.471 ± 1.461^*2^
GLB (g/L)	26.859 ± 4.745	27.582 ± 4.297	27.9 ± 4.183	28.053 ± 4.118*^1^
Dura(years)	11(8,20)	10(4,14.75)^&1^	9(3.25,11.75)^##1^	7(3,12)**^1^
Age (years, M ± SD)	13.212 ± 7.794	10.151 ± 7.109	9.504 ± 7.738	8.758 ± 7.728
PT(s)	10.575 ± 0.688	10.603 ± 0.729	10.629 ± 1.575	10.778 ± 1.672
TT(s)	17.221 ± 0.892	17.417 ± 1.056	18.11 ± 8.154	18.082 ± 7.864
Fib(g/L)	3.101 ± 0.859	3.140 ± 1.208	3.175 ± 0.959	3.348 ± 1.042*^1^
ACR(mg/g)	20(10.6)	20(10, 60)	30(10,60)	30(19.205,80.623)**^1^
ALB (g/L, x ± s)	39.259 ± 3.771	40.691 ± 4.922	41.729 ± 9.304	44.332 ± 35.321*^1^
PLT(109/L)	240.82 ± 63.491	235.88 ± 71.075	239.44 ± 60.774	243.77 ± 65.182
Hcy(µmol/L)	12.350 ± 5.918	12.534 ± 5.101	12.576 ± 5.422	14.443 ± 5.944*^1^
ALT (U/L)	16.75(13.05,20.925)	18.2(13.5,24.8)	17.9(13.5,26.3)^#1^	17.75(13.925,33.575) **^1^
GCT (U/L)	18(14.275,33.33)	20.5(15.8,26.8)	23.1(15.8,35.2)	26.45(20.025,42.175) **^1^
CREA(µmol/L)	65.2(56.375,73.125)	68.8(60.8,78.2)	68.7(57.9,79.6)	75.55(59.025,96.2) **^1^
UA(µmol/L)	292.35(235.175,353.125)	291.5(222.5,347.8)	318.1(262.3,399.8) ^##1^	333.65(274.775,395.575) **1
Cysc(mg/L)	0.915(0.768,1.050)	0.87(0.75,1.09)	0.93(0.75,1.09)	0.965(0.81,1.293) **^1^
LDL-C(mmol/L)	2.495(1.835,2.997)	2.73(2.09,3.33)	2.84(2.33,3.42) ^##1^	2.76(2.073,3.385)
HDL(mmol/L)	1.175(0.998,1.33)	1.10(0.91,1.37)	1.11(0.96,1.32)	1.03(0.893,1.196) **^1^
TG(mmol/L)	0.99(0.768,1.348)	1.23(0.93,1.71)^&&1^	1.51(1.19,2.39) ^##1^	1.7(1.113,2.893) **^1^
Fins(µU/mL)	3.57(2.145,7.393)	7.92(5.7,9.7)	11.51(7.79,14.88) ^##1^	18.965(13.01,26.828) **^1^
FBG(mmol/L)	7.1(5.298,10.323)	8.19(6.33,9.89)	8.94(6.94,11.51) ^##1^	8.56(6.758,11.093) *^1^
eGFR(ml/min/1.73m^2^, M, IQR)	94.691(84.539,104.335)	92.820(84.68,98.778)	90.837(78.294,101.239) ^#1^	89.045(64.019,100.785) **^1^
AIP	−0.0351(−0.218,0.085)	0.476(−0.144,0.221) &&^1^	0.138(−0.006,0.374) ^##1^	0.202(0.023,0.476) **^1^
TyG	8.695(8.154,9.124)	8.975(8.674,9.391) ^&&1^	9.381(8.873,9.923) ^##1^	9.385(8.834,9.821) **^1^
HOMAIR	1.447(0.574,2.854)	2.822(2.014,4.017)	4.435(2.862,6.824)^##2&2^	6.660(4.931,11.653)^##2&3^
SII	479.503(342.742, 691.400)	412.942(302.122, 666.029)	467.007(320.218, 666.029)	513.726(367.479, 685.333)

Data are expressed as mean ± standard deviation or median with interquartile range for continuous variables and as percentages for categorical variables. One-way analysis of variance (ANOVA) was used for intergroup comparisons

*Dura*: Diabetes duration, β2-MG: beta 2-microglobulin: *GLB*: globulin, *PT*: prothrombin time, *TT*: thrombin time, *Fib*: fibrinogen, *ACR*: urinary albumin/creatinine ratio, *ALB*: albumin, *PLT*: platelet, *Hcy*: homocystine, *ALT*: alanine aminotransferase, *GGT*: glutamyl transferase, *CREA*: creatinine, *UA*: uric acid, *Cysc*: cystatin C, *LDL*-*C*: low-density lipoprotein cholesterol, *HDL*: high-density lipoprotein, *TG*: triglyceride, *Fins*: fasting insulin, *FBG*: fasting plasma glucose, *eGFR* estimated glomerular filtration rate, *AIP* atherogenic index of plasma, *TyG* triglycerides-glucose, *HOMA*-*IR*: homeostatic model assessment for insulin resistance, *SII*: systemic immune-inflammatory index

Significance between groups was evaluated using ANOVA, group 3 versus groups 0, 1, and 2, *P < 0.05, **P < 0.01; group 2 versus groups 0 and 1, ^#^*P < *0.05, ^##^*P *< 0.01; group 1 versus group 0, &*P *< 0.05, &&*P* < 0.01

## Correlation between FCP and metabolic factors

 FCP level was positively correlated with the TG level (*r* = 0.202), TyG index (*r* = 0.247), AIP (*r* = 0.285), UA level (*r* = 0.248), and CREA level (*r* = 0.234) (all *P* **< 0.001)**, but negatively correlated with HDL level (*r* = − 0.166), eGFR (*r* = − 0.184), and duration of diabetes (*r* = − 0.167) (all *P* **< 0.001)**. Of note, FCP level was not significantly correlated with FBG (*r* = 0.056) and LDL-C (*r* = 0.575) levels (both *P* > 0.5) (Table 2).

**Table 2:** Correlation between FCP and metabolic or other factors

FCP	FBG	TG	Fins	HDL	LDL-C	UA	Fib	CREA	eGFR	Dura
*r*	0.056	0.202**	0.382**	−0.166**	0.023	0.248**	0.64	0.234**	−0.184**	−0.167**
*P*	0.165	0.001	0.001	0.001	0.575	0.001	0.118	0.001	0.001	0.001
FCP	AIP	TyG								
*r*	0.285**	0.247**								
*P*	0.001	0.001								

*FCP*: fasting C-peptide, *TG*: triglyceride total, *FBG*: fasting plasma glucose, *Fins*: fastinginsulin, *HDL*: high-density lipoprotein, *LDL*-*C*: low-density lipoprotein cholesterol, *UA*: uric acid, *Fib*: fibrinogen, *CREA*: creatinine, *eGFR*: estimated glomerular filtration rate, *AIP*: atherogenic index of plasma, *TyG*: triglycerides-glucose

*P < 0.05, **P < 0.01

## Univariate regression and stratified analyses

**Fig. 1:**
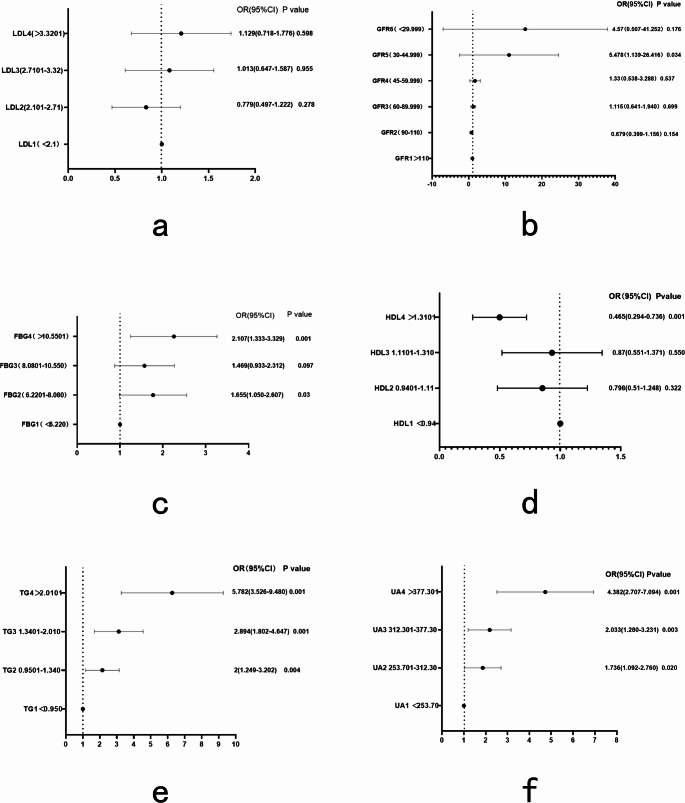
Stratified analysis of the effect of metabolic and other relevant factors on fasting C-peptide a) Stratified analysis of LDL-C (low-density lipoprotein cholesterol). b) Stratified analysis of eGFR (estimated glomerular filtration rate). c) Stratified analysis of FBG (fasting plasma glucose). d) Stratified analysis of HDL (high-density lipoprotein). e) Stratified analysis of TG (triglyceride total). f)

A stratified analysis was performed to determine the effects of metabolism and other relevant clinical factors on high FCP levels. The quartile stratification analysis indicated that for each unit increase in TG and UA levels, there was a significant increase in the corresponding FCP level (TG: odds ratio [*OR*], 5.782; 95% confidence interval [*CI*], 3.526–9.480; *P* < 0.001, and UA: *OR*, 4.382. 95% *CI*, 2.707–7.094; *P* < 0.001). TG and UA levels were identified as the most sensitive metabolic indicators of FCP level. A stratified analysis of eGFR showed that, compared with Stage 1, there was a significant increase in serum FCP levels only when eGFR decreased to 3.045 mL/min/1.73 m^2^ (*OR*, 5.486; 95% *CI*, 1.139–26.416; *P* = 0.034). Both FBG2 (6.2201–8.080 mmol/L) and FBG4 (> 10.5501 mmol/L) were associated with an elevated FCP level (*OR*, ≤ 2.107; 95% CI, 1.333–3.329; *P* = 0.001); however, different LDL-C levels had no statistically significant effect on FCP level (*OR*, 1.129; 95% *CI*, 0.718–1.776; *P* = 0.598). Age also had no significant effect on FCP levels (*OR*, 1.199; 95% *CI*, 0.726–1.982; *P* = 0.479), nor did sex (Fig. 1 and Supplementary Material).

### Multivariate regression analysis of factors affecting FCP

After adjusting for eGFR, FBG, HDL, and UA, TG level remained an independent risk factor for an increased FCP level (*OR*, 1.223. 95% *CI*, 1.044–1.433; *P* = 0.013), and UA level was secondary to TG level (OR, 1.005) (Tables 3 and 4)

**Table 3:** Multivariate regression analysis of the metabolic and other factors affecting FCP. Univariate and multivariate regression analyses were performed to determine the risk factors for increased FCP, when adjusted for eGFR, FBG, HDL, UA, and TG

	*P*	OR	95%CI	
eGFR	**0.035**	0.990	0.981	0.999
FBG	**0.006**	1.077	1.022	1.136
HDL	**0.038**	0.533	0.294	0.965
UA	**0.000**	1.005	1.003	1.007
TG	**0.013**	1.223	1.044	1.433
coefficient	**0.241**	0.412		

*eGFR*: estimated glomerular filtration rate, *FBG*: fasting blood glucose, *HDL*: high-density lipoprotein, *UA*: uric acid, *TG*: triglyceride, OR*:* odds ratio, *CI*: confidence interval

**Table 4:** The aArea under curve of FBG,UA, and TG for predicting higher FCP level

	B	SE	*p*	VIF	F	*R* ^2^	Durbin-Watson
coefficient	0.795	0.342	0.020		19.788	0.090	1.839
FBG	0.029	0.022	0.205	1.021			
TG	0.229	0.062	0.000	1.120			
UA	0.004	0.001	0.000	1.104			

*FBG*: fasting plasma glucose, *UA*: uric acid, *TG*: triglyceride total, *AUC*: area under curve: *CI*: confidence interval

### ROC curve analysis to determine the effects of high FCP levels

**Fig. 2:**
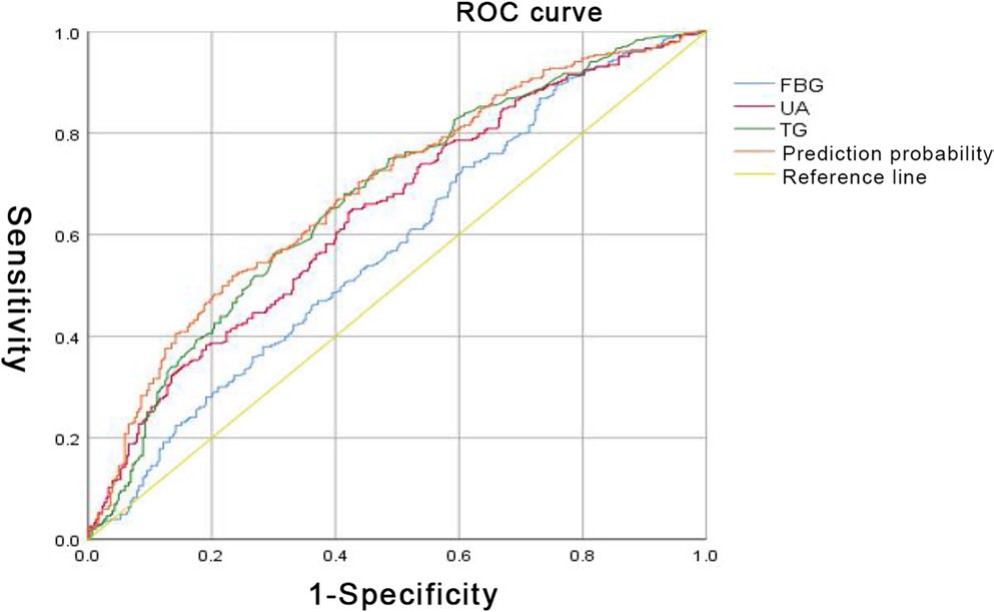
ROC curve analysis of TG, FBG, and UA on FCP FBG, fasting plasma glucose; UA, uric acid; TG, triglyceride total; ROC, receiver operating characteristic

As shown in Fig. 2, TG level was the most sensitive metabolic factor, with an AUC, specificity, sensitivity, and cutoff value of 0.671 (95% *CI*, 0.628–0.713), 0.576, 0.683, and 1.243, respectively. For FBG level, AUC, specificity, sensitivity, and cutoff value were 0.577 (95% *CI*, 0.531–0.622), 0.27, 0.868, and 5.92, respectively. For UA level, AUC, specificity, sensitivity, and cutoff value were 0.643 (95% *CI*, 0.599–0.686), 0.579, 0.644, and 305.55, respectively. The combined predictive probability AUC for these three variables was 0.686 (95% *CI*, 0.644–0.728) and the specificity, sensitivity, and cutoff value were 0.766, 0.518, and 0.527, respectively (Fig. 2 and Supplementary Materials). Linear regression was performed based on UA and TG levels, and the resultant fitting formula was 1.052 + 0.240 TG + 0.004 UA (Table 5).

**Table 5:** Based on FBG,TG, and UA levels for FCP fitted equation

	AUC	*p*	95%CI	Specificity	Sensitivity	Cutoff Value
FBG	0.577	0.001	0.531–0.622	0.27	0.868	5.92
UA	0.643	0.001	0.599–0.686	0.579	0.644	305.55
TG	0.671	0.001	0.628–0.713	0.576	0.683	1.243
Prediction probability	0.686	0.001	0.6440–0.728.6440.728	0.766	0.518	0.527

*FBG*: fasting plasma glucose, *TG*: triglyceride total, *UA*: uric acid, Fitted Equations: 0.795 + 0.029 FBG + 0.229 TG + 0.004 UA.

## Discussion

To the best of our knowledge, this is the first study aimed at elucidating the role of FCP in patients with T2DM. TG level, TyG index, and AIP were found to be significantly different between FCP quartile groups, while FCP level was linearly correlated with these values. Stratified and ROC curve analyses further confirmed that FCP level was associated with the development of lipid metabolism disorders and target organ damage, particularly based on TG levels.

Although the origin of T2DM is IR, it is only when β-cell failure leads to impaired insulin and secretion of C-peptide that fasting and postprandial hyperglycemia occur. Therefore, assessing the insulin secretory capacity of a patient is crucial for optimizing the treatment of diabetes. Actively functioning C-peptide serves as an indicator for evaluating insulin secretory capacity [23]. C-peptide can function as an endogenous antioxidant, and when combined with insulin, can prevent microvascular dysfunction induced by hyperglycemia. C-peptide can also function independently of insulin, targeting multiple tissues to exert various biological functions. The potential mechanisms of C-peptide in patients with T2DM and its chronic complications, however, have yet to be elucidated. The effects of C-peptide on cardiovascular risk may be bidirectional, with beneficial effects at a low level [24]. A higher level of FCP is reportedly associated with increased cardiovascular events and mortality, and it appears to be a promising biomarker for the prediction of microvascular changes in metabolic diseases, showing a strong correlation with peripheral microcirculation [25, 26]. These results indicate that FCP measurements have additional beneficial effects in preventing and improving vascular lesions and visceral complications in patients with diabetes [27]. C-peptide also enhances the production of nitric oxide, thereby reducing vascular resistance and promoting vasodilation [28]. Huang et al. assigned patients to four groups (Q1, Q2, Q3, and Q4) based on their FCP level, and observed that the incidence of DKD was lowest in the Q3 group (1.71 ≤ C-peptide < 2.51 ng/mL) [29]. The present study revealed that FCP level is positively correlated with the urine protein-to-creatinine ratio and serum UA level, but negatively correlated with eGFR. When eGFR was < 45 mL/min/1.73 m^2^, FCP levels increased linearly with declining eGFR, which is partially consistent with previous studies [30]. Future basic and clinical studies on C-peptide replacement therapy should consider baseline C-peptide level and prioritize monitoring the post-treatment level, to determine the optimal range for maintaining ideal fasting and postprandial C-peptide levels [31].

Lipotoxicity, a factor in the progression of DKD, is strongly associated with excessive fat production due to IR in patients with T2DM. The present study revealed that FCP level was positively correlated with TG level, TyG index, and AIP, with statistically significant differences between FCP level quartiles (*P* < 0.01). The increase in TG level was closely related to that in FCP level, with a 5.8-fold increase in FCP level for every one standard deviation increase in TG level, when TG level was >2.010, compared to a TG level < 0.950. Additionally, a 1.786-fold increase was observed in TG level for every one standard deviation increase in FCP level, when FCP level was >3.591, compared with an FCP level < 1.653. This study showed no correlation between FCP and LDL-C levels, and LDL stratification analysis indicated no statistically significant difference in FCP level across the groups, suggesting that further research is needed. Previous studies have shown that FCP levels are associated with atherosclerotic progression, although the specific mechanism behind this progression is unclear. The AIP, a sensitive indicator of atherosclerosis, had a significant positive correlation with the various FCP level quartile groups. The TyG index, a sensitive clinical indicator of IR, was positively correlated with FCP levels, suggesting that C-peptide supplementation may actually exacerbate metabolic disorders in patients with T2DM. Studies in patients with T2DM have indicated that an increase in serum FCP levels increased the risk of early atherosclerosis [32]. Wu et al. reported that FCP level in patients with IR was associated with AIP, while HOMA-IR was unrelated [33]. This discrepancy may be because C-peptide promotes TyG index- and AIP-related processes. Exogenous supplementation with C-peptide reduced glomerular hyperfiltration and albuminuria in animal models and patients with type 1 diabetes, and C-peptide has been proposed as a potential therapeutic agent for prevention of vascular damage in these patients. In the lungs of diabetic mice, the use of osmotic pumps to supplement C-peptide inhibited the production of reactive oxygen species and the activation of transglutaminase, which weakened hyperglycemia-induced pulmonary fibrosis and the expression of fibrosis-related proteins [34]. Additionally, C-peptide can inhibit cell apoptosis and tissue damage in renal tissue in high-glucose conditions, thereby providing renal protection [35]. A previous meta-analysis showed low FCP levels increased blood lipid levels and promoted lipid deposition, significantly increasing the incidence of coronary heart disease and cerebral infarction [36]. Higher concentrations of C-peptide, therefore, may exacerbate inflammation and atherosclerosis in obese patients with T2DM, consistent with our findings [37]. A previous meta-analysis (including 16 observational studies, eight cohort studies, and seven cross-sectional studies) showed that an association between C-peptide and increased cardiovascular events was only observed in cross-sectional studies [38].

Some reports have also suggested that C-peptide has a proinflammatory effect, and that the SII, an easily accessible and sensitive biomarker of systemic inflammation, reflects this inflammatory state. The present study showed no correlation between FCP and SII, and no significant difference existed in SII level between the FCP groups, suggesting that FCP may not directly participate in the inflammatory pathological mechanism. However, the effects of FCP on fibrinogen and platelets were neither relevant nor statistically significant, which is consistent with previous studies. Additionally, we found no correlation between FCP and FBG (*P* = 0.165), with a stratified analysis revealing that FBG only promoted C-peptide release at a normal glucose levels (6.22–8.08 mmol/L; *OR*: 1.655). A study involving 1,565 patients with T2DM from the Veterans Diabetes Trial showed that baseline FCP levels were negatively correlated with the risk of severe hypoglycemia [39]. The absolute value of FCP level was related to the associated blood glucose level and renal function [40]. The relationship between FCP and blood glucose levels should be further studied.

New evidence supports a dose-response association between low FCP levels and low muscle mass, indicating that FCP may be a potential biomarker of low muscle mass in patients with T2DM [41]. In contrast, patients with diabetes and high C-peptide levels are considered to have IR, for which metformin and GLP-1 receptor agonists are effective treatments [42]. It has been suggested that SGLT-2 inhibitors should be prioritized for patients with T2DM who have low FCP levels, given their influence on FCP [43].

The present study revealed that FCP plays a role in patients with T2DM by participating in TG-associated metabolic disorders. Supplemental C-peptidemay therefore not be beneficial for delaying the progression of T2DM and its complications. Despite extensive research, our understanding of FCP in T2DM remains at an early stage and requires further investigation.

The strengths of this study include a sufficiently large sample size and the representation of hospitalized patients. To enhance accuracy, most C-peptide measurements were obtained from oral glucose tolerance tests. However, the limitations of the study require that the results be carefully interpreted. The cross-sectional design prevents the establishment of causal relationshipsand future studies with larger cohorts and more precise measurements are needed to clarify these associations. Prospective clinical studies are particularly necessary to determine the impact of FCP on diabetes complications.

## Supplementary Information

Below is the link to the electronic supplementary material.

Supplementary Material 1 (DOCX. 23.8 KB)

## Data Availability

No datasets were generated or analyzed during the current study.
