# Supplementary material for: Involvement of C-peptide in the progression of type 2 diabetes mellitus through triglyceride-centered lipid metabolism
Source: Hormones (Athens). 2025 Nov 25;25(1):129–37. doi: 10.1007/s42000-025-00737-0 (PMC13013344; doi:10.1007/s42000-025-00737-0)
Supplement: Supplementary file 1 — Supplementary Material 1 (DOCX. 23.8 KB) [file 42000_2025_737_MOESM1_ESM.docx]

**Involvement of C-peptide in the Progression of Type 2 Diabetes Mellitus Through Triglyceride-centered Lipid Metabolism**

**HuiFang Li^1ID^, ZhaoMing Zhu^1^**

**Table 1** Effect of gender on fasting C-peptide

| Various | B | SE | Wald | *P-value* | OR | 96%CI | |
| --- | --- | --- | --- | --- | --- | --- | --- |
| gender(male) | -.074 | .164 | .207 | .649 | .928 | .674 | 1.279 |
| coefficient | .029 | .108 | .073 | .787 | 1.030 |  |  |

**Table 2** FCP in the non-DKD group and the DKD group

|  | No-DKD group  N=317 | DKD group  N=290 | *P-value* | F |
| --- | --- | --- | --- | --- |
| FCP | 2.77934±2.078136 | 2.97203±.110858 | 0.232 | 0.026 |

**Table 3** According to the TG grouping, the comparison between the two groups

| various | TG＜1.848 | TG＞1.8481 | t/z/x^2^ | *P-value* |
| --- | --- | --- | --- | --- |
| SII | 663.068±983.162 | 597.48±456.889 | 0.855 | 0.393 |
| AIP | 92.968（83.357,101.061） | 93.982（80.415,105.304） | -18.264 | 0.000 |
| HOMAIR | 6.874±29.987 | 7.675±14.855 | -0.363 | 0.717 |
| TyG | 8.848(8.477,9.156) | 9.905(9.513,10.324) | -16.914 | 0.000 |
| FBG | 7.910(6.070,9.950) | 8.670(6.685,11.295) | -3.058 | 0.002 |
| Fin | 8.49(4.64,14.54) | 11.97(7.14,17.29) | -4.224 | 0.000 |
| FCP(ng/mL) | 2.74(1.5,3.16) | 3.17(2.265,4.555) | -7.24 | 0.000 |
| HDL | 1.185±0.282 | 1.040±0.359 | 5.327 | 0.000 |
| LDL-C | 2.671±0.907 | 2.953±0.905 | 3.515 | 0.000 |
| UA | 299.5（238.2,357.3） | 352.8（276.55,417.150） | -6.309 | 0.000 |
| eGFR | 71.118±31.693 | 75.560±27.425 | -1.643 | 0.101 |

**Table 4** Stratified analysis of FCP on the impact of high TG

| Various | OR | 95%CI | *P-value* | B | SE | Wald |
| --- | --- | --- | --- | --- | --- | --- |
| FCPF |  |  | 0.001 |  |  | 40.851 |
| FCPF(1) | 2.333 | 1.274-4.272 | 0.006 | 0.847 | 0.309 | 7.538 |
| FCPF(2) | 3.818 | 2.129-6.846 | 0.001 | 1.340 | 0.298 | 20.221 |
| FCPF(3) | 5.963 | 3.345-10.630 | 0.001 | 1.786 | 0.295 | 36.651 |
| coefficient |  |  |  | -1.946 | 0.245 | 62.952 |

**Table 5** FCP is one of the risk factors for high TG

| Various | OR | 95%CI | *P-value* | B | 标准误差 | 瓦尔德 |
| --- | --- | --- | --- | --- | --- | --- |
| Fib | 0.963 | 0.798-1.164 | 1.164 | -0.037 | 0.096 | 0.149 |
| UA | 1.006 | 1.004-1.008 | 1.008 | 0.006 | 0.001 | 30.432 |
| HMOAIR | 1.000 | 0.991-1.009 | 1.009 | 0.000 | 0.005 | 0.002 |
| FCP | 1.225 | 1.105-1.357 | 1.357 | 0.203 | 0.052 | 14.944 |
| coefficient | 0.035 |  | 0.001 | -3.346 | 0.489 | 46.797 |

**Table 6** AIP, and TyG predict the performance of C-peptides

| various | AUC | SE | *P-value* | 95%CI | |
| --- | --- | --- | --- | --- | --- |
| AIP | .675 | .022 | .000 | .633 | .718 |
| TyG | .665 | .022 | .000 | .623 | .708 |

**Table7** Correlation analysis between C-peptide, insulin and blood glucose

| FCP | Fins | FBG |
| --- | --- | --- |
| *r* | 0.384** | 0.061 |
| *P* | 0.001 | 0.134 |
| Fins |  |  |
| *r* |  | 0.065 |
| *P* |  | 0.112 |
